# Heat Stress Reduces Intestinal Barrier Integrity and Favors Intestinal Glucose Transport in Growing Pigs

**DOI:** 10.1371/journal.pone.0070215

**Published:** 2013-08-01

**Authors:** Sarah C. Pearce, Venkatesh Mani, Rebecca L. Boddicker, Jay S. Johnson, Thomas E. Weber, Jason W. Ross, Robert P. Rhoads, Lance H. Baumgard, Nicholas K. Gabler

**Affiliations:** 1 Department of Animal Science, Iowa State University, Ames, Iowa, United States of America; 2 Department of Animal and Poultry Sciences, Virginia Polytechnic Institute and State University, Blacksburg, Virginia, United States of America; Universidad Pablo de Olavide, Centro Andaluz de Biología del Desarrollo-CSIC, Spain

## Abstract

Excessive heat exposure reduces intestinal integrity and post-absorptive energetics that can inhibit wellbeing and be fatal. Therefore, our objectives were to examine how acute heat stress (HS) alters intestinal integrity and metabolism in growing pigs. Animals were exposed to either thermal neutral (TN, 21°C; 35–50% humidity; n = 8) or HS conditions (35°C; 24–43% humidity; n = 8) for 24 h. Compared to TN, rectal temperatures in HS pigs increased by 1.6°C and respiration rates by 2-fold (*P*<0.05). As expected, HS decreased feed intake by 53% (*P*<0.05) and body weight (*P*<0.05) compared to TN pigs. Ileum heat shock protein 70 expression increased (*P*<0.05), while intestinal integrity was compromised in the HS pigs (ileum and colon TER decreased; *P*<0.05). Furthermore, HS increased serum endotoxin concentrations (*P = *0.05). Intestinal permeability was accompanied by an increase in protein expression of myosin light chain kinase (*P*<0.05) and casein kinase II-α (*P* = 0.06). Protein expression of tight junction (TJ) proteins in the ileum revealed claudin 3 and occludin expression to be increased overall due to HS (*P*<0.05), while there were no differences in claudin 1 expression. Intestinal glucose transport and blood glucose were elevated due to HS (*P*<0.05). This was supported by increased ileum Na^+^/K^+^ ATPase activity in HS pigs. SGLT-1 protein expression was unaltered; however, HS increased ileal GLUT-2 protein expression (*P = *0.06). Altogether, these data indicate that HS reduce intestinal integrity and increase intestinal stress and glucose transport.

## Introduction

Both humans and livestock are susceptible to high thermal loads that can cause acute, chronic, and lethal illness due to heat stress-related pathologies. In 2003, approximately 50,000 Europeans died during an intense heat-wave [1], [2]. More recently, about 11,000 people succumbed to heat stress in Moscow during an abnormally hot 2010 summer [3]. Besides cooling and rehydration, there are few standard medical procedures to treat heat stroke and mortality for patients admitted to hospitals is thought to be >30% [4]. Moreover, heat stress is also important for animal agriculture as it is estimated to costs the US swine industry over $300 million annually and cost global animal agriculture tens of billions of dollars [5].

Heat-stressed mammals partition blood to the periphery in an attempt to maximize radiant heat dissipation, and this blood redistribution is supported by vasoconstriction of the gastrointestinal tract [6]. As a result, reduced blood and nutrient flow leads to hypoxia at the intestinal epithelium, which ultimately compromises intestinal integrity and function [7]. Consequently, heat-induced intestinal permeability is associated with increased blood markers of endotoxemia, hypoxia, and inflammation; all of which may contribute to multi-organ failure syndrome [8]. As the gastrointestinal tract is highly sensitive to hyperthermia, and a compromised mucosa is pivotal to the pathobiology of heat -related illness, numerous animal and cell culture models have been utilized to examined the etiology of heat-induced intestinal damage [9]–[11]. However, few studies have examined how high ambient temperatures affect intestinal function and integrity.

The mechanisms by which heat stress alters intestinal permeability are not fully understood. However, inflammation and hypoxia regulate intestinal tight junction (TJ) proteins such as occludin and claudins, along with heat shock proteins (HSP) and hypoxia-inducible factor (HIF) [12]–[14]. Additional consequences of altered intestinal permeability may include changes in nutrient digestibility and absorption across the intestinal epithelium. Therefore, our objective was to evaluate and characterize the effects of prolonged HS on intestinal integrity, metabolism, and function in growing pigs. Furthermore, we hypothesized that an acute heat-load would detrimentally alter intestinal integrity leading to augmented endotoxemia and inflammation.

## Materials and Methods

### Animals and Study Design

All procedures involving animal use and care were approved by the Iowa State University Institutional Animal Care and Use Committee. Sixteen crossbred pigs (46±6 kg BW) comprising of six gilts and ten barrows were housed in individual pens and allowed *ad libitum* access to water and feed at all times. Feed intake was measured throughout the acclimation period, and the diet consisted primarily of ground corn and soybean meal and was formulated to meet or exceed nutrient requirements [15]. After two weeks of acclimation under thermal neutral conditions, pigs were randomly assigned to one of two climatic treatments.

To evaluate the effects of an acute heat load, pigs were exposed to either thermal neutral (TN, 21°C; 35–50% relative humidity; n = 8) or HS conditions (35°C; 24–43% relative humidity; n = 8) for 24 h. Regardless of environmental treatment, all animals were fed the same diet throughout the duration of the experimental period. During the 24 h experimental period animals were monitored continuously for signs of distress. Rectal temperatures were recorded with a digital thermometer (Top care®, Waukegan, IL) and respiration rates (breaths/min) calculated with a stopwatch. Pigs were moved into HS conditions in six blocks beginning at 0800 or 1100 h over three days. For each block, respiration rate, rectal temperature, and feed intake were measured every four hours during each 24-h period. Body weights were recorded on all animals at 0 h and immediately prior to sacrifice (i.e. 24 h). Blood was obtained while the animals were restrained (at 0 and 24 h) and immediately sacrificed using a penetrating captive bolt followed by exsanguination.

Biological samples harvested included whole tissue and mucosal scrapings from the ileum (2 m proximal from the ileal-cecal junction) and colon (1 m from the rectum). A portion of the tissue samples were snap-frozen in liquid nitrogen and stored at −80°C until further analyses. An additional fresh sample of whole ileum and colon was obtained and placed immediately into Krebs-Henseleit buffer (containing 25 mM NaHCO_3_, 120 mM NaCl, 1 mM MgSO_4_, 6.3 mM KCl, 2 mM CaCl and 0.32 mM NaH_2_PO_4_, pH 7.4) under constant aeration for transport to the laboratory and mounting into modified Ussing Chambers.

### Ussing Chamber

Intestinal tissue from the proximal ileum and colon was mounted into modified Ussing chambers (Physiological Instruments, San Diego, CA) for determining intestinal integrity and active nutrient transport. Tissue samples were pinned and placed vertically into the chambers, connected to dual channel current and voltage electrodes submerged in 3% noble agar and filled with 3 M KCl for electrical conductance. Each segment was bathed in 4 mL of Krebs-Henseleit buffer (KHBB) on both serosal and mucosal sides, and tissue was provided with a constant O_2_-CO_2_ mixture. Individual segments were clamped at a voltage of 0 mV and transepithelial electrical resistance (TER) was determined [16]. Ileal active glucose and glutamine nutrient transport were measured as previously described by Gabler and co-workers [16].

To further assess intestinal integrity, ileum and colon mucosal to serosal macromolecule transport of 4.4 kDa fluorescein isothiocyanate labeled dextran (FITC-Dextran) was used. After 20 min of stabilization, Krebs-Henseleit buffer was removed from the luminal side and 2.2 mg/mL of FITC-Dextran was added while 4 mL of KHBB was added to the acceptor side. Samples from both sides were obtained in duplicate every 20 min for 80 min. The relative fluorescence was then determined using a fluorescent plate reader (Bio-Tek, USA) with the excitation and emission wavelengths of 485 and 520 nm, respectively. Thereafter, an apparent permeability coefficient (Papp) was calculated for each treatment [17]:
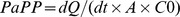
Where: dQ/dt = transport rate 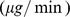; C0 = initial concentration in the donor chamber 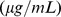; A = area of the membrane (cm^2^).

### Blood Glucose and Endotoxin Analysis

Blood glucose was analyzed using an i-STAT® (Abbott Point of Care, Princeton, NJ) machine with the CG8+ cartridge. Blood from the 0 and 24 h sampling was obtained using lithium heparin vacutainer tubes. Serum endotoxin concentrations were determined using a commercially available kit validated for use in our laboratory. Briefly, endotoxin concentrations were determined in triplicate using a recombinant Factor C (rFC) endotoxin assay with a 1/1000 dilution factor for porcine serum samples (PyroGene® Recombinant Factor C Endotoxin Detection System, Lonza, Walkersville, MD). The procedure was conducted in 96-well microplates and fluorescence was measured at time 0 and after 1 h incubation at 37°C. The plates were then read under fluorescence using a Synergy 4 microplate reader (Bio-Tek, Winooski, VT) with excitation/emission wavelengths of 380/440 nm. Relative fluorescence unit (RFU) was determined and concentration of endotoxin was interpolated from the standard curve. Values were calculated by subtracting the endotoxin concentration at time zero of the study from the concentration at time of sacrifice.

### Myeloperoxidase Activity (MPO)

To assess immune cell infiltration into the intestinal tract due to HS, whole ileum tissue MPO activity was measured using a modified method previously described [18]. Briefly, tissue samples were homogenized in 0.5% hexadecyltrimethylammonium bromide (HTAB) in potassium phosphate buffer (PPB; pH 6.0) and then freeze-thawed and vortexed three times. Samples were then centrifuged for 15 min at 10,000×g. The resulting supernatant was transferred to a new tube and the remaining pellet was again suspended in 500 µL of 0.5% PPB+HTAB. The resuspended pellet was freeze-thawed and homogenized 2× and 500 µL was transferred to a new tube. Samples were then centrifuged again at 10,000×g for 15 min and the supernatant was collected. The final supernatant was mixed with o-dianasidine dihydrochloride and 0.005% hydrogen peroxide. One unit of MPO activity was expressed as the amount of MPO needed to degrade 1 µmol of hydrogen peroxide/min/mL. Absorbance was read at 460 nm for 10 min reaction time and absorbance was calculated on a milliliter sample/milligram protein basis.

### Na^+^/K^+^ ATPase Activity

Ileal mucosal scrapings were homogenized in sucrose buffer (pH 7.4) consisting of: 50 mM sucrose, 1 mM Na_2_EDTA, and 20 mM tris base and centrifuged at 1000×g for 10 min for protein extraction. Protein extracts were separated into 5 aliquots: two for water, two for ouabain, and one for bicinchoninic acid (BCA) protein analysis. Proteins with either MQ H_2_O or 20 mM ouabain were pre-incubated for 15 min with Na^+^/K^+^ ATPase reaction buffer (pH 7.0; 2000 mM NaCl, 100 mM KCl, 50 mM MgCl_2_ and 250 mM HEPES) and then incubated for 45 min after addition of fresh 105 mM ATP to start the reaction. After 45 min the reaction was terminated using ice-cold 50% trichloroacetic acid. Samples were centrifuged at 1500×g for 10 min to obtain the final product which was present in the supernatant [19]. Lastly, samples were analyzed for the presence of inorganic phosphate using the Molybdovanadate method [20] and assessed in triplicate at a wavelength of 400 nm using a Synergy 4 microplate reader (Bio-Tek, Winooski, VT). Specific Na^+^/K^+^ ATPase activity was determined by the difference in inorganic phosphate (P_i_) production from ATP in the presence of absence of ouabain (specific Na^+^/K^+^ ATPase inhibitor). Unspecific phosphate hydrolysis was correlated by measuring P_i_ freed in the absence of protein suspension.

### Western Blotting

Whole cell protein from ileal mucosal scrapings was extracted in a PBS +1% Triton X-100 buffer with protease and phosphatase inhibitors and used for a majority of western blot analysis. For TJ measurement, proteins from ileum mucosal scrapings were fractionated into cytosolic and membrane portions as previously described [21]. Briefly, tissues were homogenized in a lysis buffer containing 1% Triton X-100, 100 mM NaCl, 10 mM HEPES pH 7.6, 2 mM EDTA with protease and phosphatase inhibitors. Samples were centrifuged at 15,000×g for 30 min and the triton-soluble (cytosolic) supernatant was collected. The pellet was ultrasonicated in triton buffer with 1% SDS and centrifuged at 15,000×g for 5 min to give the triton-insoluble (membrane) fraction. Tissue homogenates were separated by SDS (10–15%) polyacrylamide gel electrophoresis (SDS-PAGE). Gels were run under reducing conditions and transferred to nitrocellulose membranes. Membranes were blocked for 1 h in 5% non-fat dry milk (NFDM) in TBST (1× TBS, 0.1% Tween-20). Membranes were then blocked in primary antibody with 5% NFDM in TBST overnight. After blocking in primary antibody (HSP70, HIF 1-α, MLCK, GLUT 2, SGLT-1, C-Src, CK II-α, Claudin 1, Claudin 1, Occludin, MCT, and GAPDH; Table S1) membranes were incubated in secondary antibody for one hour. For detection, Supersignal® West Pico Chemiluminescent Substrate was used (Thermoscientific, Waltham, MA). Membranes were imaged using FOTO Analyst® Luminary/FX® (Fotodyne Inc, Hartland, WI). Band densities were quantified by densitometry using TotalLab Quant (Total Lab®, Newcastle Upon Tyne, UK). Bands were standardized to the density of GAPDH and represented as a ratio of each protein to GAPDH.

### Inflammation Measures

Equal amounts of ileal mucosal scraping tissue protein (100 µg) and serum were analyzed for interleukin-1β (IL1B) and IL-8 concentrations using a porcine-specific ELISA (DuoSet® Porcine IL1, catalog number DY681 and DY535, respectively, R&D systems, Minneapolis, MN, USA) per the manufacturer’s instructions. Further, serum tumor necrosis factor (TNF)-α was measured also using a commercially available ELISA kit (Quantikine® Porcine TNF-α, catalog number PTA00, R&D systems, Minneapolis, MN, USA).

### Digestive Enzyme Activities

Activities of maltase, sucrase, and lactase were analyzed in ileal mucosal scrapings using a modified method of Dahlqvist [22], and liberated glucose was measured using the glucose oxidase method. L-alanine aminopeptidase activity was analyzed using a modified method of Roncari and Zuber [23], utilizing p-nitroaniline as a substrate.

### Statistics

All data were statistically analyzed using the PROC MIXED procedure of SAS version 9.2 (SAS Inst. Inc. Cary NC). The model included a fixed effect of treatment (TN vs. HS) as well as a random effect of sex and experimental block if significant. For hourly measurements (body temperatures, respiration rates and feed intake) each animal’s respective parameter was analyzed using repeated measures with an auto-regressive covariance structure and time as the repeated effect. Baseline or time 0 data was used as a covariate. All data are reported as LS means and differences considered significant if *P*<0.05 and a tendency if *P*<0.10.

## Results

### Phenotypic Response to Heat Stress

**Figure 1:**
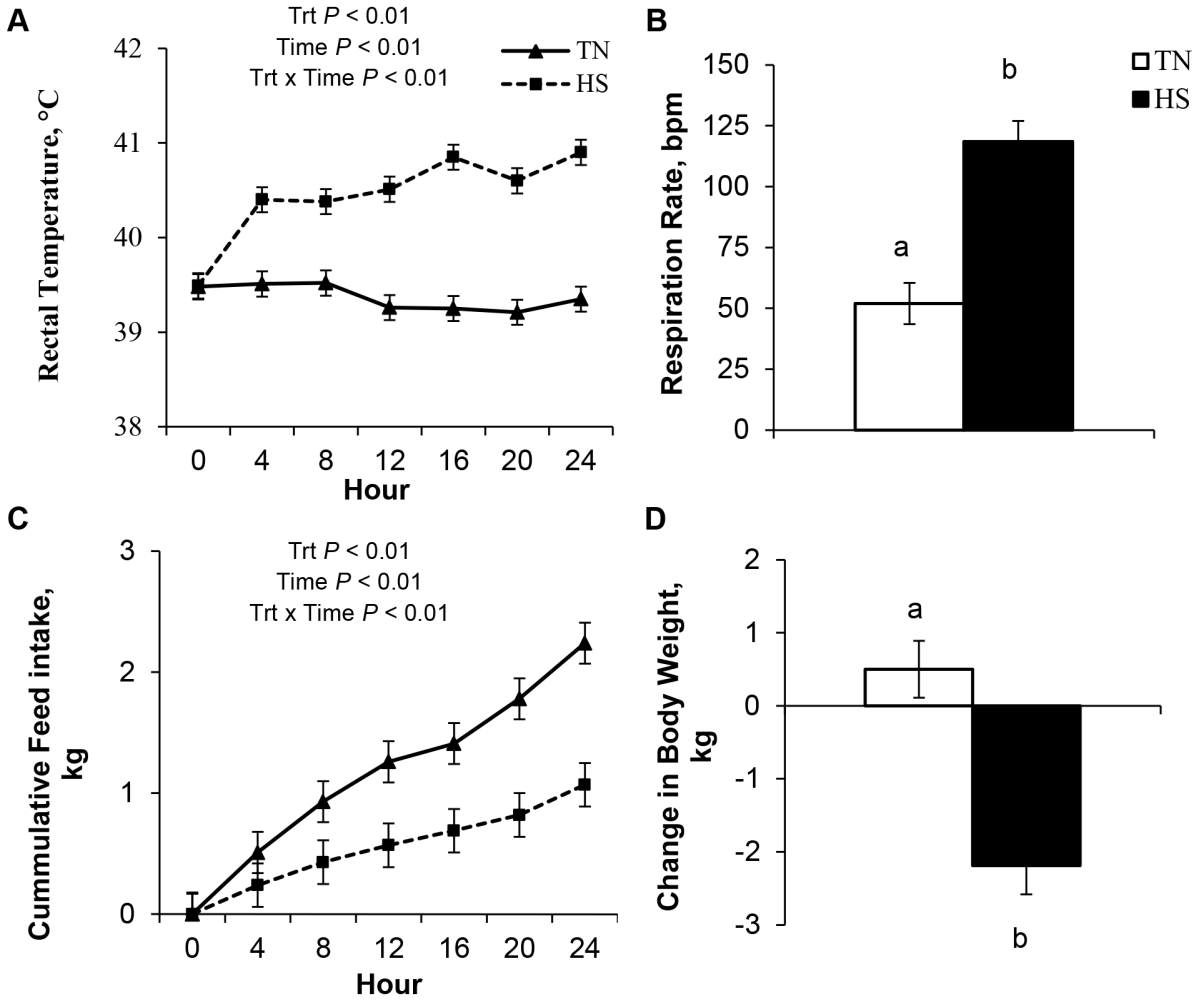
The effects of 24 hours of either constant thermal neutral conditions (TN; 21°C) or heat stress conditions (HS; 35°C) on a) rectal temperature, b) respiration rates, c) cumulative feed intake, and d) change in body weight in growing pigs. ^a,b^ *P*<0.05, n = 8/trt.

Heat-stressed pigs had an immediate and overall increase in rectal temperatures (39.3 vs. 40.9°C, *P*<0.01, Figure 1a) and an approximate 2-fold increase in overall respiration rate compared to the TN controls (52 vs. 119 bpm, *P*<0.05, Figure 1b). During HS, cumulative feed intake decreased (53%; *P*<0.01, Figure 1c) and heat-stressed pigs lost BW (−2.2 kg; *P*<0.05, Figure 1d) while TN pigs gained BW (0.5 kg).

### Intestinal Integrity

**Figure 2:**
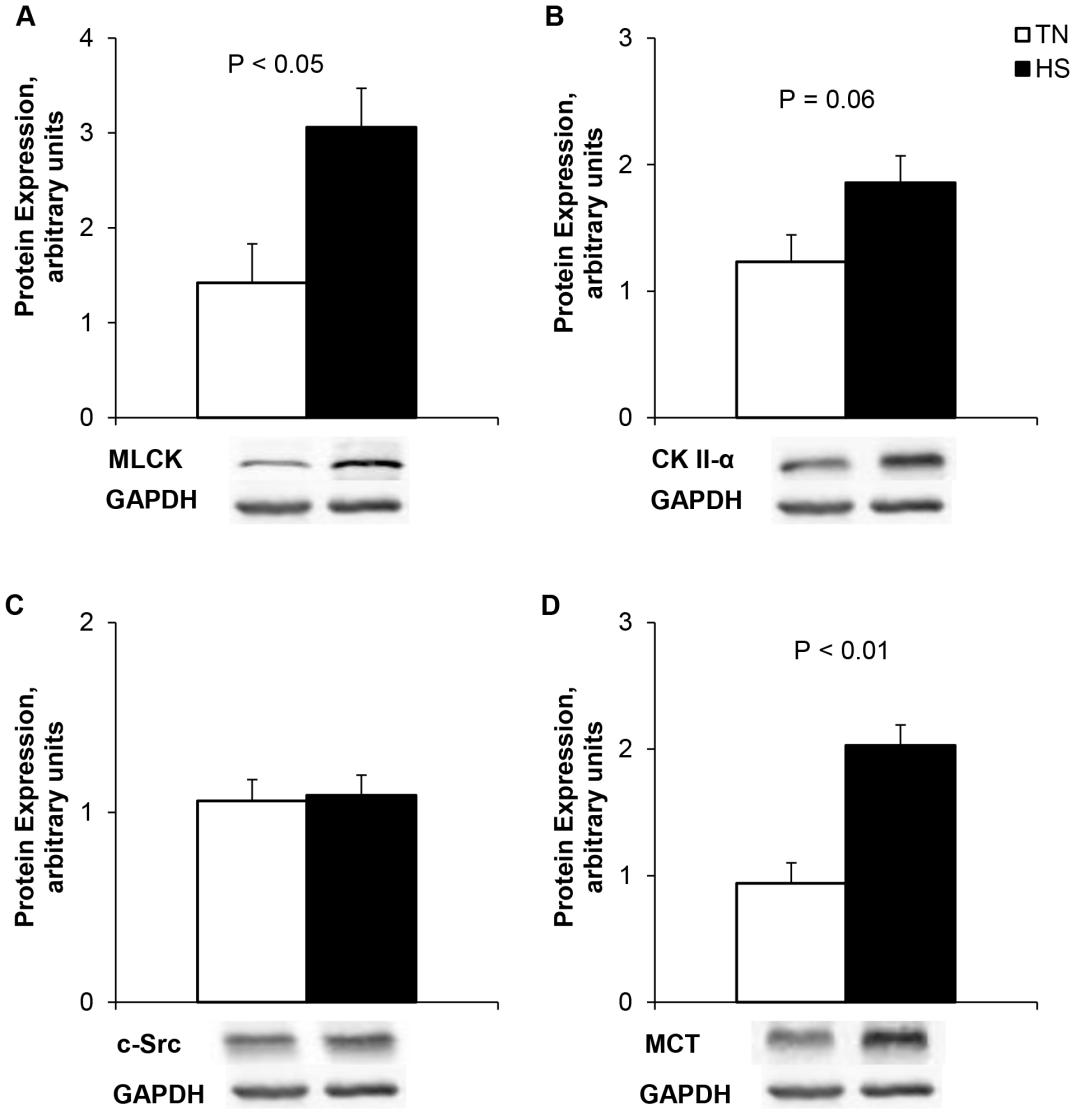
Heat stress augments ileum protein expression of TJ protein regulatory machinery; a) Myosin light chain kinase (MLCK), b) Casein kinase (CK) II-α, c) c-Src, and d) Mast cell tryptase (MCT) in growing pigs. Pigs were exposed for 24 hours to either constant thermal neutral conditions (TN; 21°C) or heat stress conditions (HS; 35°C). n = 8/trt.

**Figure 3:**
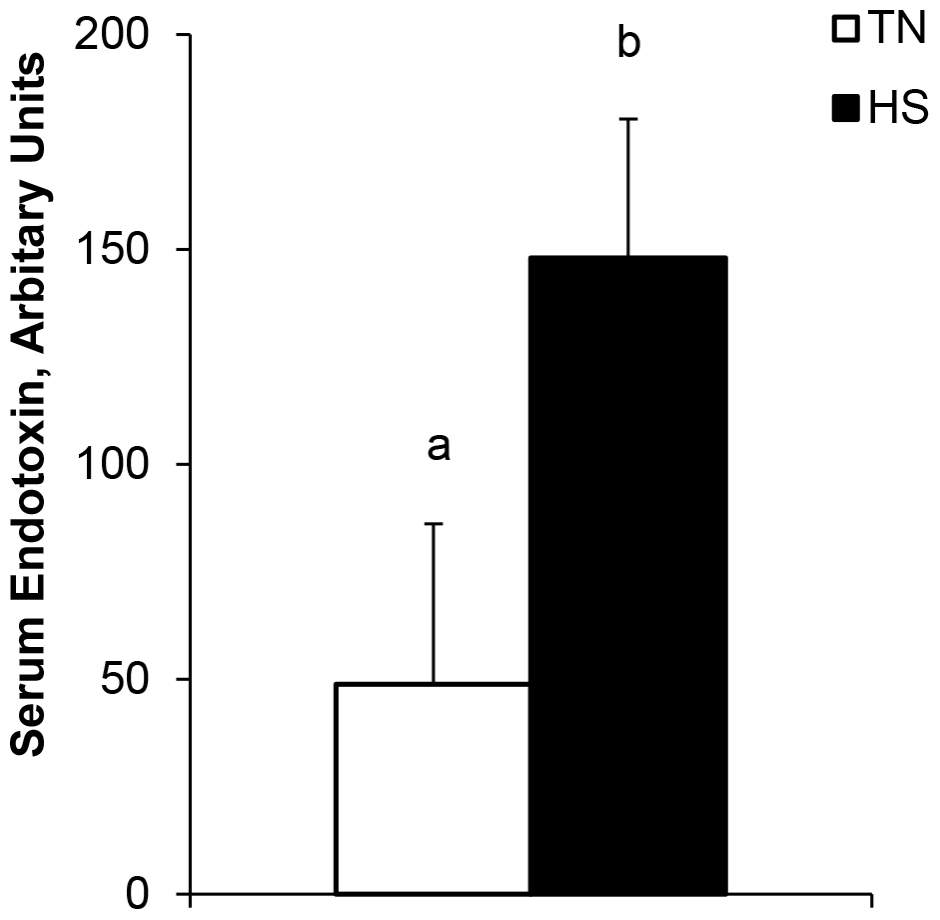
Heat stress conditions (HS; 35°C) increases serum endotoxin compared to pigs reared in thermal neutral conditions (TN; 21°C) for 24 hours. ^a,b^ *P*<0.05, n = 8/trt.

**Figure 4:**
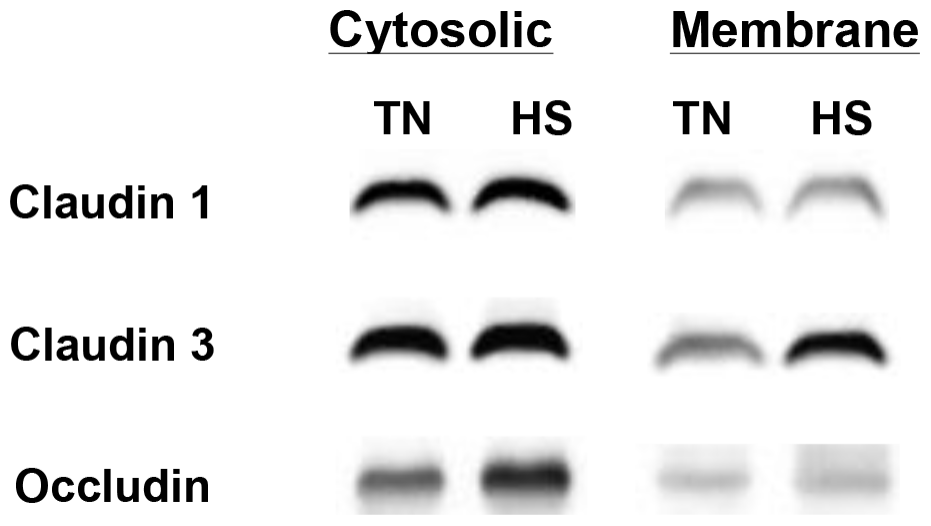
Heat stress conditions (HS; 35°C) alters expression and localization of TJ proteins compared to pigs reared in thermal neutral conditions (TN; 21°C) for 24 hours. Supplemental figure in support of Table 2.

Ileum and colon TER decreased 52 and 24%, respectively in heat-stressed pigs (*P*<0.05, Table 1). Furthermore, the apparent permeability coefficient (transport of the macromolecule, FITC-dextran) was markedly elevated in the ileum and colon (119 and 472%, respectively; *P*<0.05, Table 1) due to HS. Protein expression of ileum mucosal MLCK was elevated during HS (116%; *P*<0.05, Figure 2a). There was a tendency (*P* = 0.06; Figure 2b) for HS to increase expression of ileum CK II-α, however c-Src expression was unchanged due to temperature (*P*>0.10, Figure 2c). Circulating endotoxin concentrations also increased 200% in HS pigs compared to TN counterparts (*P*<0.05, Figure 3). There were no differences (*P*>0.10) detected in cytosolic or membrane fraction protein expression of claudin 1 (Table 2, Figure 4). Total claudin 3 was upregulated (*P*<0.05) due to HS and this difference was solely due to an increase in the membrane fraction (42%; *P*<0.05). Occludin protein expression was increased in the total fraction (*P*<0.05, Table 2, Figure 4), however, differed from claudin 3, in that the increase was solely in the cytosolic fraction due to HS. (44%; *P*<0.01, Table 2, Figure 4).

**Table 1:** Ileum and colon intestinal integrity is compromised in pigs reared in heat stress (HS) conditions compared to thermal neutral (TN).

Parameter	Environment^a^		*P-value*
	TN	HS	
Ileum TER^b^, Ω×cm^2^	182.3±17.4	87.7±18.6	<0.01
Colon TER^b^, Ω×cm^2^	133.0±7.2	101.6±8.3	<0.01
Ileum APP^c^, µg/mL/min/cm	3.61±0.93	7.92±1.08	<0.01
Colon APP^c^, µg/mL/min/cm	2.74±3.55	15.67±3.55	0.02

a Pigs were exposed for 24 hr to either thermal neutral (TN; 21°C) or heat stress (HS; 35°) conditions. Mean ± S.E.M, n = 8/trt.

b Transepithelial electrical resistance (TER).

c Apparent permeability coefficient of FITC-Dextran transport (APP).

**Table 2:** Ileum tight junction protein expression from pigs reared in thermal neutral or heat stress conditions for 24 h.

	Cytosolic Fraction^b^		Membrane Fraction^c^		Cyto/Mem Ratio^e^		*P*-value			
Parameter	TN^a^	HS^a^	TN^a^	HS^a^	TN^a^	HS^a^	Cyto^b^	Mem^c^	Total^d^	Ratio^e^
Claudin 1	1.01±0.12	1.10±0.12	1.09±0.19	1.28±0.18	1.06±0.16	0.71±0.14	0.62	0.47	0.15	0.14
Claudin 3	1.12±0.06	1.21±0.06	1.00±0.09	1.42±0.09	1.12±0.07	0.87±0.07	0.34	<0.01	0.06	0.02
Occludin	1.14±0.11	1.64±0.12	0.45±0.07	0.59±0.08	2.82±0.32	2.93±0.32	0.01	0.22	0.02	0.82

a Pigs were exposed for 24 hr to either thermal neutral (TN; 21°C) or heat stress (HS; 35°) conditions. Mean ± S.E.M, n = 8/trt.

b Cytosolic Fraction – Protein expression relative to a control sample on each gel, Arbitrary units.

c Membrane Fraction – Protein expression relative to a control sample on each gel, Arbitrary units.

d Sum of cytosolic and membrane protein expression densitometry data.

e Ratio of cytosolic/membrane fraction.

### Intestinal Metabolism

**Figure 5:**
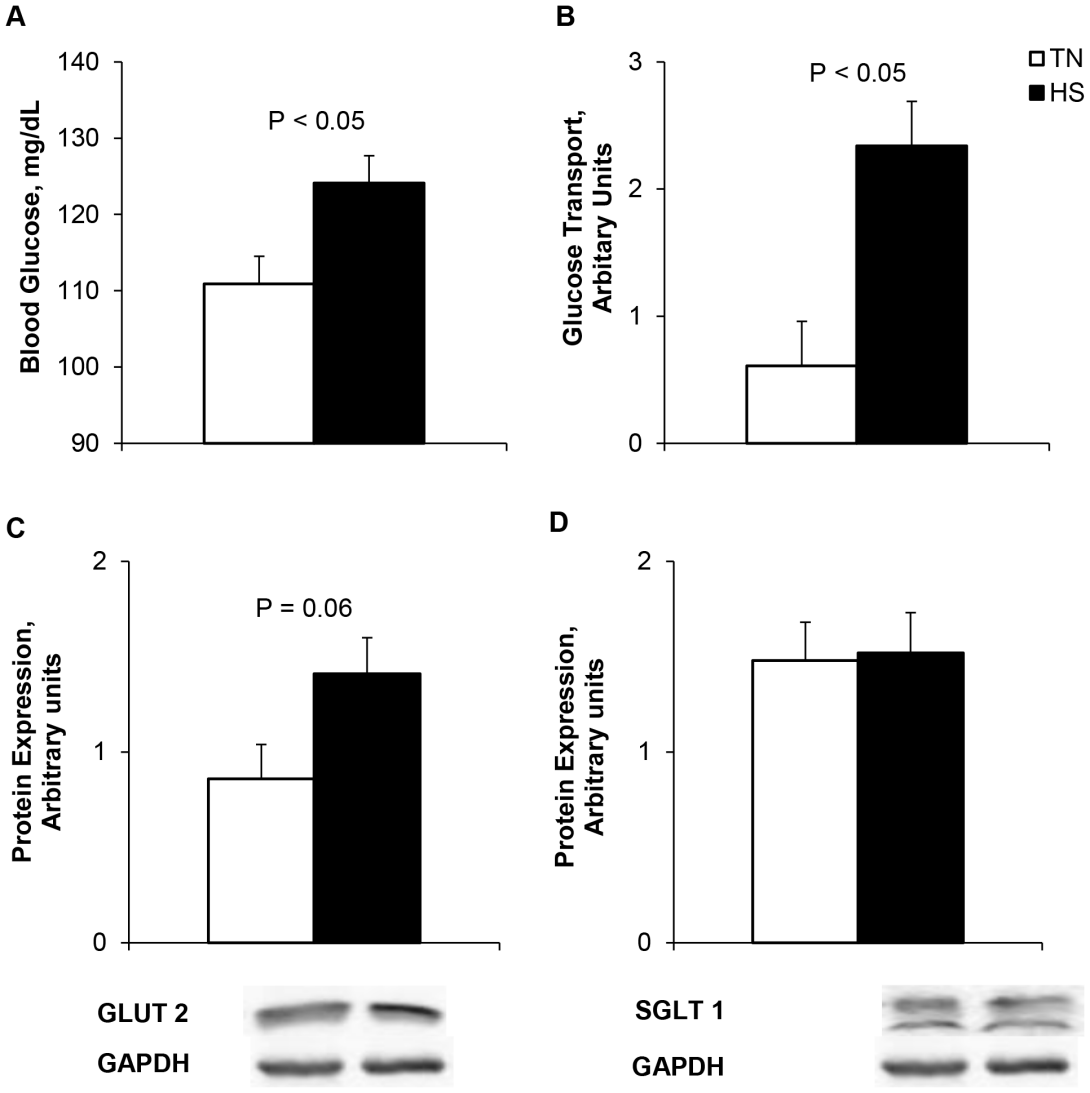
The effects of 24 hours of either constant thermal neutral conditions (TN; 21°C) or heat stress conditions (HS; 35°C) on a) blood glucose, b) active glucose transport, c) GLUT-2 expression, and d) SGLT-1 expression in growing pigs. n = 8/trt.

**Figure 6:**
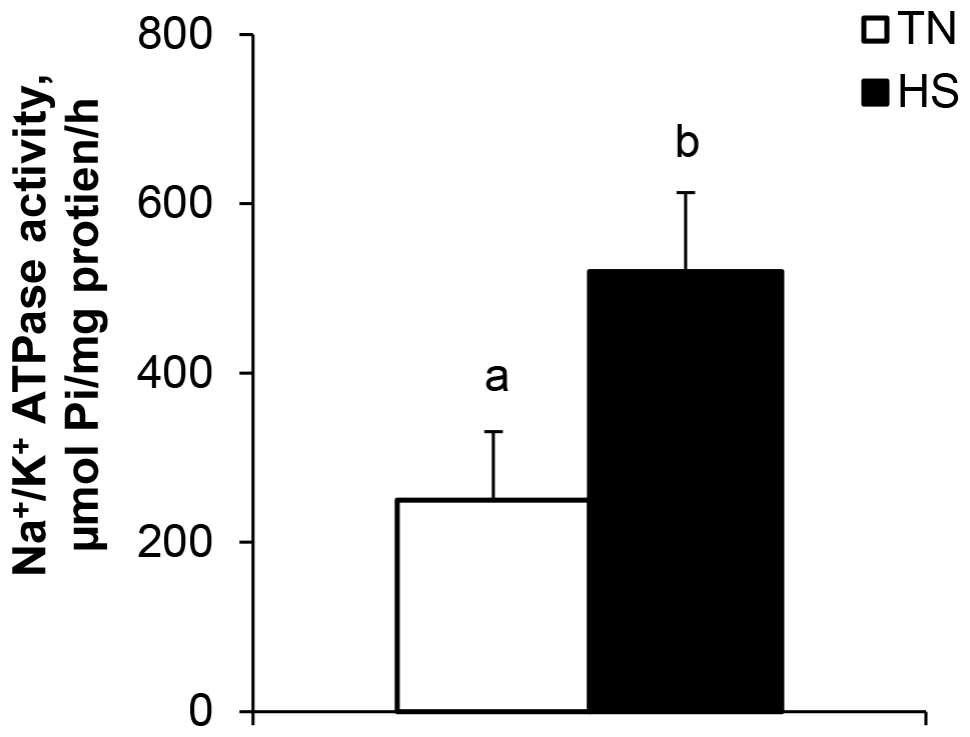
Heat stress conditions (HS; 35°C) increase Na^+^/K^+^ ATPase activity compared to pigs reared in thermal neutral conditions (TN; 21°C) for 24 hours. ^a,b^ *P*<0.05, n = 8/trt.

After 24 h of an acute heat load, HS pigs had increased blood glucose (12%; *P*<0.05, Figure 5a). Ileum active glucose transport activity was increased (283%; *P*<0.05; Figure 5b) during HS, and HS tended to increase glutamine transport (*P = *0.09, Table 3). HS tended to increase GLUT-2 protein expression (*P* = 0.06, Figure 5c). However, no changes were detected in SGLT-1 protein abundance (*P*>0.05, Figure 5d). Ileal Na^+^/K^+^ ATPase activity was increased due to HS (109%; *P*<0.05, Figure 6) while sucrase and maltase activities were decreased by HS (30 and 24%, respectively; *P*<0.05, Table 3). Lactase activity did not differ between treatment groups (*P = *0.37, Table 3). There were no differences in ileal mucosal aminopeptidase N activity (*P = *0.17, Table 3).

**Table 3:** The effects of 24 h of thermal neutral (TN) or heat stress (HS) conditions on intestinal glutamine transport and digestive enzymes in growing pigs.

Parameter	Environment^a^		*P-value*
	TN	HS	
Ileum glutamine transport, Arbitrary Units	1.02±0.31	1.93±0.38	0.09
Lactase activity^b^, µmol/min/g protein	2.21±0.47	1.62±0.44	0.37
Sucrase activity^b^, µmol/min/g protein	43.5±4.2	30.5±4.2	0.05
Maltase activity^b^, µmol/min/g protein	246.8±12.1	187.0±12.1	<0.01
Aminopeptidase N, µmol/min/g protein	1.62±0.17	1.95±0.16	0.17

a Pigs were exposed for 24 hr to either thermal neutral (TN; 21°C) or heat stress (HS; 35°) conditions. Mean ± S.E.M, n = 8/trt.

b Liberated glucose.

### Immune and Stress Response

**Figure 7:**
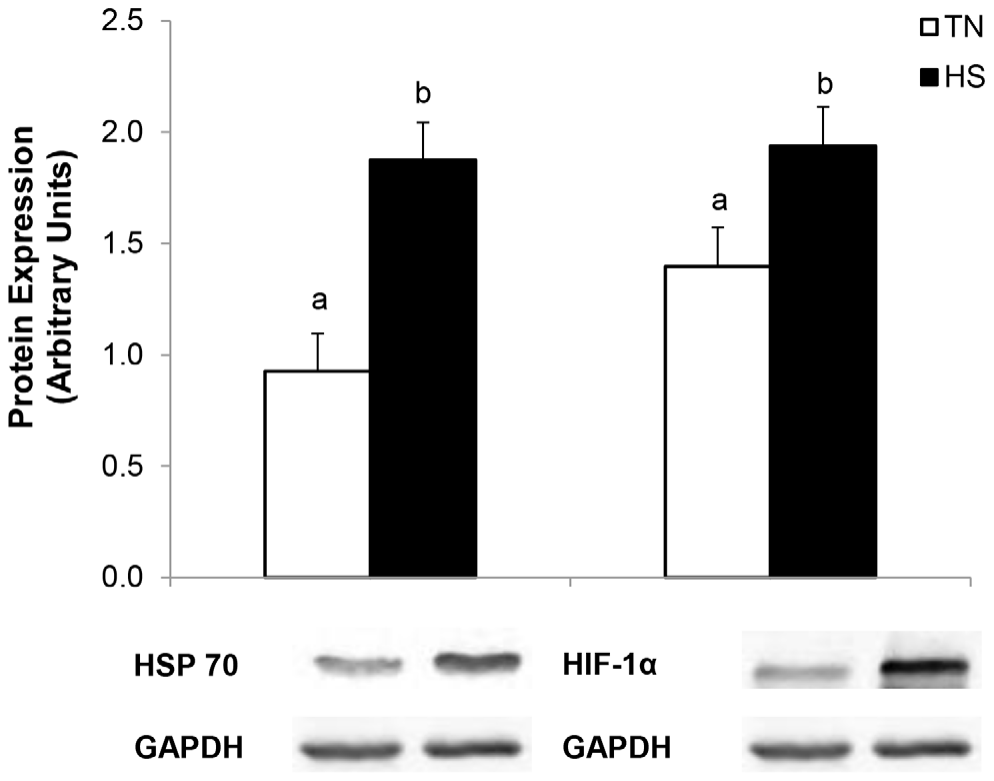
The effects of 24 hours of either constant thermal neutral conditions (TN; 21°C) or heat stress conditions (HS; 35°C) on a) mucosal HSP70 expression or b) mucosal HIF-1α protein expression in growing pigs. ^a,b^ *P*<0.05, n = 8/trt.

As expected, HS increased mucosal HIF-1α (139%, *P*≤0.05, Figure 7) and HSP70 protein expression (201%, *P*<0.01, Figure 7). There were no differences observed in ileal mucosal IL1B and IL-8 concentrations, however there was a HS-induced decrease in serum IL-8 (56%; *P*<0.01, Table 4), serum TNF-α (*P*<0.05) and a tendency for a decrease in serum IL1β (62%; *P* = 0.11, Table 4). Ileal MPO activity was increased in HS pigs compared to TN counterparts (147%; *P*<0.05, Table 4). Protein expression of ileal mast cell tryptase (MCT) was increased due to HS (116%; *P*<0.01, Figure 2d).

**Table 4:** Ileum and serum inflammatory responses are differentially modulated after 24 h of thermal neutral (TN) or heat stress (HS) conditions in growing pigs.

Parameter	Environment^a^		*P-value*
	TN	HS	
*Ileum*			
Interleukin 1β, pg/µg protein	144.0±17.0	124.5±17.0	0.43
Interleukin 8, pg/µg protein	418.2±18.1	376.4±17.0	0.12
Myeloperoxidase Activity, U/mg protein	9.0±0.9	13.3±0.7	<0.01
*Serum*			
Interleukin 8, pg/mL	1237±118	548±118	<0.01
Interleukin 1β, pg/mL	217.1±54.8	82.5±54.8	0.11
Tumor necrosis factor-α, pg/mL	53.1±3.17	42.5±3.17	0.03

a Pigs were exposed for 24 hr to either thermal neutral (TN; 21°C) or heat stress (HS; 35°) conditions. Mean ± S.E.M, n = 8/trt.

## Discussion

Exposure to high ambient temperatures can impose considerable health and physiological stress-related problems to humans and animals, and the gastrointestinal tract is one of the main organs affected [6]. By design, our HS protocol resulted in marked hyperthermia as evidenced by elevated rectal temperatures and respiration rates. Another immediate effect of HS was the decrease in feed intake, and this reduced appetite is presumably a strategy to minimize metabolic heat production. Decreased feed intake during HS was expected as this is a highly conserved response amongst species [24], [25]. However, it is important to note that nutrient restriction can lead to alterations in intestinal function, transport, morphology, and this may increase the risk of developing bacterial sepsis [26]. Heat-stressed pigs also lost a significant amount of body weight in 24 h and this demonstrates that our acute model was an incredibly catabolic event. This may be explained by the fact that pigs are extremely susceptible to HS because they lack functional sweat glands and produce a large amount of metabolic heat [27]. However, the loss of body weight could be partially explained by a combination of reduced feed intake, increased basal metabolic rate and a presumed increase in urination. Although, no diarrhea was observed due to HS and water consumption was not recorded, we believe our HS pigs were well hydrated as crudely assessed by blood hematocrit values which were not different from the TN control pigs (data not shown).

Heat stress repartitions blood to the periphery which results in reduced blood flow to the intestinal epithelium and may lead to hypoxia, ATP depletion, oxidative and nitrosative stress, as well as apoptosis [6]. ATP depletion and osmotic stress is further amplified by an increase in ion pump activities that are a significant source of cellular and whole body energy expenditure [28]. During heat stress, cells also become more permeable to sodium and require more energy to maintain osmolyte homeostasis, membrane potential and active nutrient transport [29], [30]. As such, this likely explains the increase in intestinal Na^+^/K^+^ ATPase activity observed in our HS model. We also observed increased cellular stress in the form of increased protein expression of HIF 1-α and HSP70 during HS. Under hypoxic conditions, HIF-1-α is rapidly upregulated to support many aspects of cell survival [31]. On the other hand, heat shock proteins, are a diverse family of proteins that are important in the stress response as they act as chaperones and housekeepers to provide protection and recovery to proteins which are misfolded, unfolded, or otherwise altered due to elevated temperatures [32]. Specific to thermal biology, HSP70 is rapidly upregulated 2–4 h after heat exposure [9]. In addition, HSP70 mediates responses to endotoxin induced cytokine production and may interfere with nuclear factor (NF)-κB transcription, thus diminishing or disrupting the inflammatory response [33]. Altogether, these data indicate that 24 h of HS induces a localized stress response to the gastrointestinal tract.

Inflammation and pro-inflammatory cytokines [34] as well as hypoxia [35] regulate or disrupt TJ’s and reduce intestinal integrity. In the current study, HS increased circulating endotoxin, endotoxin transport, and intestinal permeability. As a result, increased permeability can lead to endotoxemia and systemic inflammation [8]. This reduction in intestinal integrity is likely in part due to decreased resistance across the intestinal epithelium, as evidenced by a reduced TER during HS. The decrease in TER agrees with previous HS models in cell culture and rodents [9], [10].

TJ proteins such as zonula occludin, claudins, and occludins re-distribute or re-localize during times of stress [14], [36], [37]. ZO-1 is thought to primarily regulate paracellular permeability in the intestine, while the role of occludin in paracellular permeability is still not fully understood [38]. In the current study we observed an overall (membrane plus cytosolic) increase in claudin 3 and occludin protein expression due to HS. Our occludin data agrees with previous 24 h intestinal HS models in which occludin protein is upregulated [9], [39]. Upregulation of these TJ proteins may indicate a barrier enhancement effect during HS in an attempt to compensate for increased permeability. This notion is supported by data indicating heat-induced expression of HSPs are required for occludin upregulation [9]. Another possibility is that the occludin detected is not functionally bound to ZO-1 and TJ complexes. Interestingly, the distribution of TJ proteins differed, in that claudin 3 protein expression was upregulated more in the membrane fraction, while occludin was upregulated more in the cytosolic or detergent insoluble fraction.

Re-distribution of TJ proteins has previously been shown due to oxidative stress [40] in a c-Src kinase dependent manner [41]. Phosphorylation of occludin by multiple kinases and phosphatases is thought to contribute to TJ regulation and modification [42]. The Src-family kinases are thought to be involved in TJ assembly and intestinal integrity. Casein kinase II-α also plays a key role in occludin phosphorylation and acts as an important regulator of ZO-1, claudin-1, and claudin-2 proteins. TJ protein complexes dissociate and lead to impaired barrier function upon CKII-α activation [38]. In the current study we have shown HS to increase ileum CK II-α expression, indicating another mechanism through which TJ disruption of the intestinal epithelium may occur. Interestingly, no differences in c-Src expression were observed due to HS. c-Src has been shown to directly bind and phosphorylate occludin, thus diminishing its ability to bind to zonula occludins [42]. Epithelial cells also consist of an actin cytoskeleton and contraction of this cytoskeleton is necessary to maintain cell motility. Regulation of the cytoskeleton is largely mediated by MLCK and post-translational modifications to myosin light chain [14]. Increased expression and activation of MLCK and subsequent phosphorylation of myosin light chain has been observed during HS and increases intestinal permeability [43]. This agrees with our data, as HS increased MLCK protein expression. Interestingly, MLCK is known to be activated by oxidative stress, hypoxia and HIF 1-α [12]. Moreover, it has been established that Na+-glucose cotransporter, SGLT1, activation in Caco-2 monolayers increases tight junction permeability [44]. Thus, this suggests that the enhanced glucose transport also contribute to intestinal tight junction remodeling via MLC/MLCK activation and increased paracellular permeability [45].

Environmental stresses such as HS and hypoxia cause local and systemic inflammation in a number of human and animal models [8], [46], [47]. Myeloperoxidase activity was used as an indirect measure of ileum inflammation and more specifically, as a measure of neutrophil infiltration which produces hypochlorous acid to aid in pathogen killing. Our findings indicate that HS induced MPO activity. Interestingly, Kansagra et al., [48] reported moderate to weak correlations between MPO activity and intestinal permeability in a piglet enteric versus total parental nutrition model. Thus, suggesting a clear link between intestinal inflammation and permeability. Mast cells are activated due to an immune response and release tryptase which activates downstream signaling pathways involved in inflammation. Previous reports indicate an increase in MCT (in a model of weaning stress) which is correlated with an increase inflammation and intestinal permeability [49], [50]. Intriguingly, the pro-inflammatory cytokines IL-8, IL1B, and TNF-α were reduced in circulation and remained unchanged in the ileum epithelium. This was surprising as we hypothesized that endotoxemia and hypoxia would have increased these cytokines in our model. This discrepancy may be explained by the fact that the peak immune-febrile response may have occurred earlier than 24 h and that a down regulation of this cytokine response was measured [51]. Alternatively, the up regulation of HSP70 has been shown to reduce TLR-4 induced signaling in enterocytes [32] and thus HSP70 may be diminishing NF-κB activation and the subsequent pro-inflammatory response.

Metabolically, both HS and endotoxin induced inflammation shift post-absorptive fuel selection from oxidative phosphorylation to glycolytic metabolism, while deemphasizing fatty acid oxidation [25], [52], [53]. Our HS pigs were hyperglycemic after 24 h and this may partially be a result of increased ileum glucose transport. This data is supported by data showing nutrient absorption optimization and post-absorptive metabolism changes in HS poultry [54]. Furthermore, we and others [55] have reported an increased intestinal membrane GLUT2 protein expression that would aid in passive glucose uptake. However, we observed no differences in ileal SGLT-1 protein expression in the HS pigs, which is contrary to avian HS data [54]. Amino acid metabolism is also of interest as glutamine is a primary energy source for intestinal cells [56]. It is not clear whether the observed changes reflect increased transport or increased glutamine oxidation. Regardless, glutamine appears to play a key role in maintaining intestinal health [56] and this is likely similar during HS. Although contradictory to the glucose transport data, ileal mucosal sucrase and maltase activities were attenuated by HS (Table 3). These digestive enzyme reductions due to HS may be explained by increased epithelial sloughing or atrophy [57].

In addition, our glucose findings may also suggest possible mechanisms for cellular protection and hydration by the intestinal epithelium form HS. Intestinal SGLT-1-mediated glucose uptake has been shown to protect intestinal epithelial cells against LPS and *Giardia* induced apoptosis via targeting mitochondrial dependent and independent pathways [58], [59]. Mechanistically, this is a result of LPS induced CD14 activation of SGLT-1, independent of TLR4, that ultimately leads to cell rescue [60]. The increase in glucose transport due to HS may also be a reflection of increased water transport. Water can be co-transported along with Na = and glucose through SGLT-1 [61].

In conclusion, exposure to acute HS had reduced intestinal integrity and increased circulating endotoxin. Furthermore, intestinal glucose transport, digestive capacity and post-absorptive metabolism is adversely affected during acute HS. Further research is warranted with a pair-fed control group to elucidate differences that may be related to caloric restriction verses direct heat. Short term exposure to high ambient heat increases intestinal permeability and targets key kinases that regulate TJ complexes. Osmotic stress, hypoxia and inflammation appear all to contribute to the intestinal pathologies of heat stress.

## Supporting Information

Table S1 **Primary antibody and source information for Western blot analysis.**
(DOCX)
