# Supplementary material for: Heat Stress Reduces Intestinal Barrier Integrity and Favors Intestinal Glucose Transport in Growing Pigs
Source: PLoS One. 2013 Aug 1;8(8):e70215. doi: 10.1371/journal.pone.0070215 (PMC3731365; doi:10.1371/journal.pone.0070215)
Supplement: Table S1 — Primary antibody and source information for Western blot analysis. (DOCX) [file pone.0070215.s001.docx]

**Table S1.** Primary antibody and source information for Western blot analysis

| **Protein** | **MW, kDa** | **1° dilution** | **1° Source** | **2° dilution** |
| --- | --- | --- | --- | --- |
| Mouse anti-HSP70^a^ | 70 | 1:1000 | Novus Biologicals | 1:10,000 |
| Mouse anti-HIF-1α^b^ | 120 | 1:1000 | Thermo Scientific | 1:10,000 |
| Rabbit anti-Claudin 3 | 22 | 1:750 | Invitrogen | 1:10,000 |
| Rabbit anti-Claudin 1 | 22 | 1:750 | Invitrogen | 1:10,000 |
| Mouse anti-Occludin | 65-80 | 1:1000 | Invitrogen | 1:10,000 |
| Rabbit anti-GLUT2^c^ | 53-61 | 1:500 | Chemicon International | 1:10,000 |
| Rabbit anti-MLCK^d^ | 211 | 1:1000 | Abcam | 1:10,000 |
| Rabbit anti-SGLT1^e^ | 70-77 | 1:1000 | Chemicon International | 1:10,000 |
| Mouse anti-GAPDH^f^ | 36-38 | 1:1000 | Biochain | 1:10,000 |
| Rabbit anti-MCT^g^ | 34 | 1:1000 | Santa Cruz Biotechnology | 1:10,000 |
| Rabbit anti-c-Src^h^ | 60 | 1:500 | Santa Cruz Biotechnology | 1:10,000 |
| Mouse anti-CK II-α^i^ | 45 | 1:500 | Santa Cruz Biotechnology | 1:10,000 |

^a^ Heat shock protein 70 (HSP-70)
 ^b^ Hypoxia-inducible factor (HIF-1α)
 ^c^ Glucose transporter 2 (GLUT2)
 ^d^ Myosin light chain kinase (MLCK)

^e^ Sodium-glucose transporter 1 (SGLT1)

^f^ Glyceraldehyde-3-phosphate dehydrogenase (GAPDH)

^g^ Mast cell tryptase (MCT)
 ^h^ Cellular-sarcoma (c-Src)
 ^i^ Casein kinase II-α (CK II-α)
